# An in vitro test bench reproducing coronary blood flow signals

**DOI:** 10.1186/s12938-015-0065-x

**Published:** 2015-08-07

**Authors:** Kamil Jerzy Chodzyński, Karim Zouaoui Boudjeltia, Jacques Lalmand, Adel Aminian, Luc Vanhamme, Daniel Ribeiro de Sousa, Simone Gremmo, Laurent Bricteux, Christine Renotte, Guy Courbebaisse, Grégory Coussement

**Affiliations:** Laboratoire de Médecine Expérimentale (ULB 222 Unit), Route de Gozée 706, 6110 Montigny-Le-Tilleul, Belgium; Faculté Polytechnique de Mons, Service Fluides-Machines, Université de Mons, 53, rue du Joncquois, 7000 Mons, Belgium; Service of Cardiology, CHU Charleroi, Boulevard Zoé Drion 1, 6000 Charleroi, Belgium; Laboratory of Molecular Parasitology, IBMM, Université Libre de Bruxelles (ULB), 12 rue des Prof. Jeener et. Brachet, 6041, Gosselies, Belgium; Automatic Control Department, University of Mons, 31, Boulevard Dolez, 7000 Mons, Belgium; CREATIS, CNRS UMR 5220, INSERM U1044, UCB Lyon1, INSA Lyon, University of Lyon, 7 Av. Jean Capelle Building Blaise Pascal, 69621 Villeurbanne Cedex, France

**Keywords:** Pulsatile shear stress, In vivo measurements, Coronary arteries, In vitro test bench

## Abstract

**Background:**

It is a known fact that blood flow pattern and more specifically the pulsatile time variation of shear stress on the vascular wall play a key role in atherogenesis. The paper presents the conception, the building and the control of a new in vitro test bench that mimics the pulsatile flows behavior based on in vivo measurements.

**Methods:**

An in vitro cardiovascular simulator is alimented with in vivo constraints upstream and provided with further post-processing analysis downstream in order to mimic the pulsatile in vivo blood flow quantities. This real-time controlled system is designed to perform real pulsatile in vivo blood flow signals to study endothelial cells’ behavior under near physiological environment. The system is based on an internal model controller and a proportional-integral controller that controls a linear motor with customized piston pump, two proportional-integral controllers that control the mean flow rate and temperature of the medium. This configuration enables to mimic any resulting blood flow rate patterns between 40 and 700 ml/min. In order to feed the system with reliable periodic flow quantities in vivo measurements were performed. Data from five patients (1 female, 4 males; ages 44–63) were filtered and post-processed using the Newtonian Womersley’s solution. These resulting flow signals were compared with 2D axisymmetric, numerical simulation using a Carreau non-Newtonian model to validate the approximation of a Newtonian behavior.

**Results:**

This in vitro test bench reproduces the measured flow rate time evolution and the complexity of in vivo hemodynamic signals within the accuracy of the relative error below 5%.

**Conclusions:**

This post-processing method is compatible with any real complex in vivo signal and demonstrates the heterogeneity of pulsatile patterns in coronary arteries among of different patients. The comparison between analytical and numerical solution demonstrate the fair quality of the Newtonian Womersley’s approximation. Therefore, Womersley’s solution was used to calculate input flow rate for the in vitro test bench.

## Background

Atherosclerosis is a chronic inflammatory disease of large- and medium-size arteries [1]. Although the entire vasculature is exposed to the atherogenic effects of various systemic risk factors (e.g., dyslipidemia, smoking, hypertension, diabetes and genetic predisposition) [2–5], atherosclerotic lesions form at specific regions of the arterial tree. This behavior is associated with local variations in arterial hemodynamic properties and more particularly with the pulsatile time dependent evolution of the wall shear stress. The effects of shear stress are linked to the expression levels of atheroprotective and atherosensitizing endothelial genes [6, 7], cells need to be able to adapt to these constantly changing stimuli.

Several studies [8–10] showed that vascular endothelial cells (ECs) respond differentially to various patterns of pulsatile flow. Different EC responses can be recorded not only as a function of the time-averaged wall shear stress (WSS) but also of the wall shear stress time derivative (WSSTD), known also as the shear stress slew rate (SSSR) [8]. Also unsteady pulsatile flow induced quicker EC elongation and realignment rates as compared to steady flow, despite identical time-averaged shear stress [8]. In their review [10], Lee Stoner et al. highlighted the two main limitations of in vitro studies in this field: (1) ECs from different tissue beds respond differently to a given flow; (2) most investigators submit ECs to a single type of stimulus (mostly a constant laminar shear stress), which probably fails to accurately mimic the complex response to the in vivo signals. Therefore, additional studies comparing different pulsatile flow patterns are required to enact the different conditions and the coronary wall shear stresses encountered in vivo.

In a review article dedicated to the shear stress biology of the endothelium, Davies et al. [11] suggested future directions in this field of research. Two points caught our attention: (1) the design of in vitro experiments that critically test the dominant fluid dynamic conditions existing in vivo and address the validity of in vitro models for intact tissues; (2) the determination of the importance of wall shear stress gradients on endothelial biology and of the temporal changes occurring throughout the cardiac cycle.

Based on these suggestions this manuscript focus on the signals related to real flow conditions as well as in the design of an in vitro device capable of reproducing hemodynamic patterns of atherogenic conditions in vitro. To build this synthetic device, one needs to be able to mimic, in vitro, the exact signals encountered in the blood vessels. This ability would enable signals to be generated and applied to ECs or multisheet culture systems containing ECs and SMCs (*smooth muscle cells*) seeded in a transparent tube that would also allow observation under the microscope, recording of flow parameters and cell harvest after treatments. In order to development such device, the in vivo signal, such as blood velocity, have to be recorded and afterwards, post-processed to enable extraction of derived physical quantities, such as flow rate, WSS and WSSTD. With this goal (building an in vitro system that mimics real pulsatile blood patterns) in mind, the first objective was to accurately record real time dependent hemodynamic data. We performed in vivo measurements using a Doppler flow ComboWire^®^ (Volcano Corp.) in five patients, in order to gain knowledge of in vivo instantaneous maximal velocity of coronary arteries. In addition, the diameter of the artery at different points was measured by quantitative coronary angiography (QCA). Moreover, the hematocrit level from each patient was obtained. In order to extract additional derived physical variables, such as instantaneous flow rate, WSS and WSSTD from the in vivo measurements, these quantities were post-processed. This applied post-process consisted in analytical integration of the unsteady Navier–Stokes equation using the Womersley’s relation based on the assumption of a Newtonian fluid with constant viscosity in a straight rigid pipe. Since blood has a viscosity that varies with the shear rate [12], we used 2D axisymmetric, non-Newtonian CFD simulations (using a Carreau model) to validate the approximation of a Newtonian behavior.

Several projects are being carried out worldwide to provide a system capable mimicking, outside the human body, the circulatory system [8, 13, 14]. However, these in vitro systems mostly focus on constant and simple harmonic flow oscillations. This can be an important limitation because in vivo studies have found [8, 14] that the flow topology and the consequent shear stress do have a significant role in vascular response. Moreover, some of these experiments hardly incorporate physiological properties in their experimental conditions such as sterility, gas balance, proper pH and osmolarity as well as duration of experiment.

Lately, several investigators have tried to improve the different types of systems in order to overcome these limitations [14–16]. Two of these attempts involve [14, 16] similar systems. Both aim to reproduce as accurately as possible the effective in vivo pressure signals. They also are closed, circular systems starting and ending in the same reservoir. The third one is based on the cone-plate model. They all have their advantages and disadvantages, the most important inconvenience being that they are mostly designed for one particular selected experiment. Thus one of them [16] was built to work with ECs in 3D geometry (and involves straight pipes). The constructors focused mainly on pressure signal control. The second one [14] was built to allow researchers to work with different vessel geometries such as for example aneurysms and visualise different flows by using a PIV laser. In this case, researchers focused on simple flows. The corn-plate system [15] was designed to apply various shear stress signals on ECs attached to the plate. A 3D vessel model is also required to test medical devices such as Flow Diverted Stents. However, a pipe system requires large medium volumes which may create sterility problems. This problem was circumvented by the cone-plate system as a little mound of medium is used. Finally, none of these systems is fully automatic, they require manual adjustments, they have poor mobility and flexibility and they need an additional device to keep sterile conditions.

The system described here tries to preserve all advantages and overcome all disadvantages of the systems mentioned above. An additional key feature was to construct an universal machine capable reproducing, as accurately as possible, any in vivo flows measurements. Therefore, the research and development was focused on the control of pulsating flow rate and corresponding shear stress instead of pressure. Nevertheless, the system is designed so that in the future both pressure and flow rate would be controlled simultaneously. On top of mimicking the pulsatile flow behaviour and values recorded in vivo in the cardiovascular system, a supplementary constrain was to build a test bench easy to work with, mobile, as automatic and compact as possible and easily adaptable to work with additional supporting equipment such as PIV laser or CT scanner [17]. Downstream it should also automatically record values on demand such as flow rate, pressure, temperature and/or allow to capture images of the EC culture chamber while the system is running. All these requirements are met by the presented test bench.

## Methods

The study can be divided into three parts: (1) Measurements, including in vivo real time velocity; (2) post-processing measured data including filtering of the measured velocity and calculating derived quantities, such as flow rate, WSS and WSSTD, 3) reproduction of in vivo patient’s signal by presented developed in vitro test bench.

### Patients: in vivo instantaneous velocity measurements and filtering

**Fig. 1:**
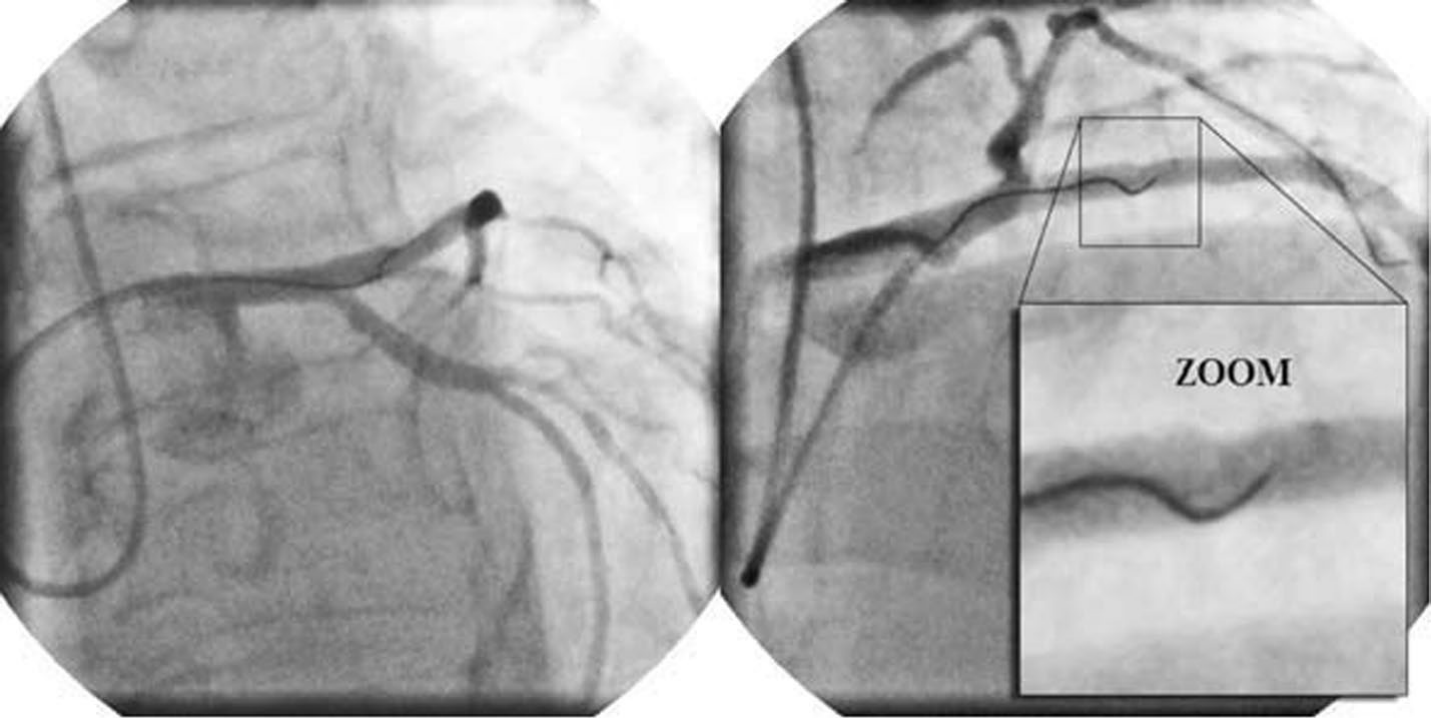
Angiographies showing the position of the wire for in vivo measurements. An angioplasty was performed (pictures took for patient 1) in the left anterior descending artery (not shown). Access was obtained via the radial artery using a 6 Fr XB 3.5 guide catheter. It was then used to insert a ComboWire^®^ allowing coronary blood velocity to be measured. Angiographies were taken from two different angles to visualize the position of the measurement point at a central position (*right*, zoom).

The patients were selected from those who needed a coronarography. The protocol number B3252006117 was approved by the Ethics Committee of OM008 of the ISPPC (Intercommunale de Santé Publique du Pays de Charleroi) Hospital and the volunteers gave their written informed consent. Coronary arteries were selected devoid of any pathology. The subjects included in the study are described in detail below:

Patient 1 was a 58-year-old male with stable angina admitted for elective percutaneous intervention (PCI) of a long and severe stenosis involving the third marginal branch. His past medical history included previous anterior myocardial infarction treated with a drug eluting stent (DES) on the left anterior descending artery (LAD), diabetes, hypertension, active smoking and dyslipidemia. Access was obtained via the right radial artery using a 6 Fr XB 3.5 guide catheter. Angiographic images from the patient are shown in Fig. 1. After completion of the third marginal angioplasty, a ComboWire^®^ was advanced into the proximal part of the LAD for coronary artery blood velocity measurements.

Patient 2 was a 44-year-old female undergoing coronary angiography for unstable angina. Her cardiac risk factors included dyslipidemia and active smoking. Access was obtained via the right femoral artery using a 6 Fr XB 3.5 guide catheter. After implantation of a bare metal stent (BMS) in the proximal LAD, a ComboWire^®^ was advanced into the proximal part of the LAD.

Patient 3 was a 63-year-old man with stable angina pectoris and a positive myocardial perfusion scan. His past medical history included insertion of a DES in the mid-left anterior descending artery, hypertension and type 2 diabetes. Access was obtained via the right radial artery using a 5 Fr XB 3.5 guide catheter. The coronary angiogram showed no significant lesion on the coronary tree. The ComboWire^®^ was placed in the intermediate artery for coronary blood velocity measurements.

Patient 4 was a 46-year-old man with dyslipidemia and previous implantation of BMSs in the proximal LAD and mid-left circumflex artery (LCX), who was undergoing control angiogram for recurrent angina. Access was obtained via the right radial artery using a 6 Fr XB 3.5 guide catheter. After successful PCI of a LCX intra-stent occlusion, the combo wire was advanced in the intermediate artery for coronary blood velocity measurements.

Patient 5 was a 63-year-old man with stable angina pectoris and a positive myocardial perfusion scan. His cardiac risk factors included dyslipidemia and type 2 diabetes. Access was obtained via the right radial artery using a 5 Fr XB 3.5 guide catheter. Coronary angiogram showed no significant coronary lesion. The ComboWire^®^ was placed in the LAD for coronary blood velocity measurements.

All patients had normal left ventricular systolic function except for patient 1 who had moderate left ventricular systolic dysfunction (ejection fraction of 45%).

For all the measurements, special care was taken to keep the tip of the wire in the center of the artery lumen and to obtain optimal measurements (Fig. 1) so that the measured velocity was maximal. The diameter of the artery at different points was measured by quantitative coronary angiography (QCA) and the values are shown in Table 1.

**Table 1:** The averaged data decomposed by Fourier series and the variability of velocities as well as periods Δ and diameters D of the arteries for all patients tested

	Patient 1	Patient 2	Patient 3	Patient 4	Patient 5
U_min_ (cm/s)	3.7 ± 3.5	5.8 ± 1.5	5.8 ± 1.6	16.3 ± 3.6	9.6 ± 2.4
U_mean_ (cm/s)	18.6 ± 4.1	22.7 ± 1.1	19.6 ± 3.2	35.7 ± 4.2	27.7 ± 5.9
U_max_ (cm/s)	32.9 ± 9.0	38.2 ± 2.3	33.2 ± 2.3	58.2 ± 10.9	41.1 ± 13.8
Period Δ (s)	1.06 ± 0.13	0.83 ± 0.06	0.81 ± 0.08	0.85 ± 0.08	0.81 ± 0.16
D (mm)	3.39	2.52	2.01	2.24	2.14

Measurements were made using a ComboMap^®^ analysis unit (Volcano Corp.), a PC-based system with an LCD color touch screen and a remote control operation. The ComboMap^®^ system provides both pressure and flow rate (fractional flow reserve (FFR) and coronary flow reserve (CFR)) and allows digital storage to the hard drive or archival storage to a CD. A Doppler flow intravascular wire (ComboWire^®^ with a diameter 0.3556 mm) was used to measure the instantaneous velocity.

**Fig. 2:**
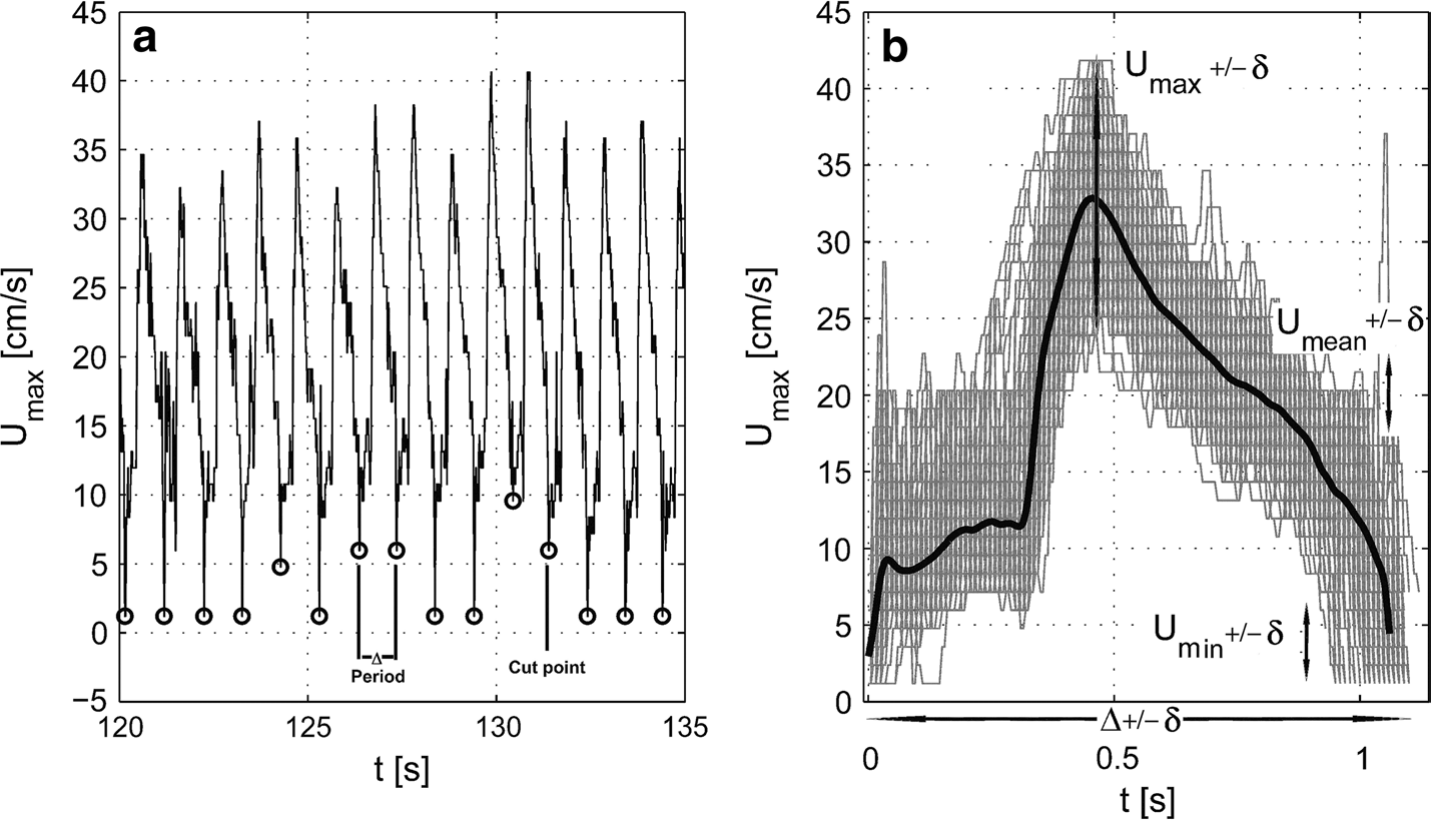
Measured coronary velocity: raw data and post-processed data. A ComboWire^®^ was used to measure coronary artery velocity signals. **a** A time window of the measurements of the coronary artery velocity signal measured in patient 1 indicating some variability from cycle to cycle. The cut points of the signal are indicated as *empty circles*. The differences between cut points present a period Δ of the heart beat. **b** Filtered coronary velocity cycles for patient 1. *Gray lines* represent superimposed filtered averaged velocity *curves* measured for all cycles in patient 1. The *block line* represents the mean filtered velocity signal. Variation in maximum velocity is indicated by the *double*-*headed arrow*. U_min_ ± δ, mean velocity; U_mean_ ± δ, maximum velocity; U_max_ ± δ as well as period of the cycle Δ ± δ (*arrows*).

**Fig. 3:**
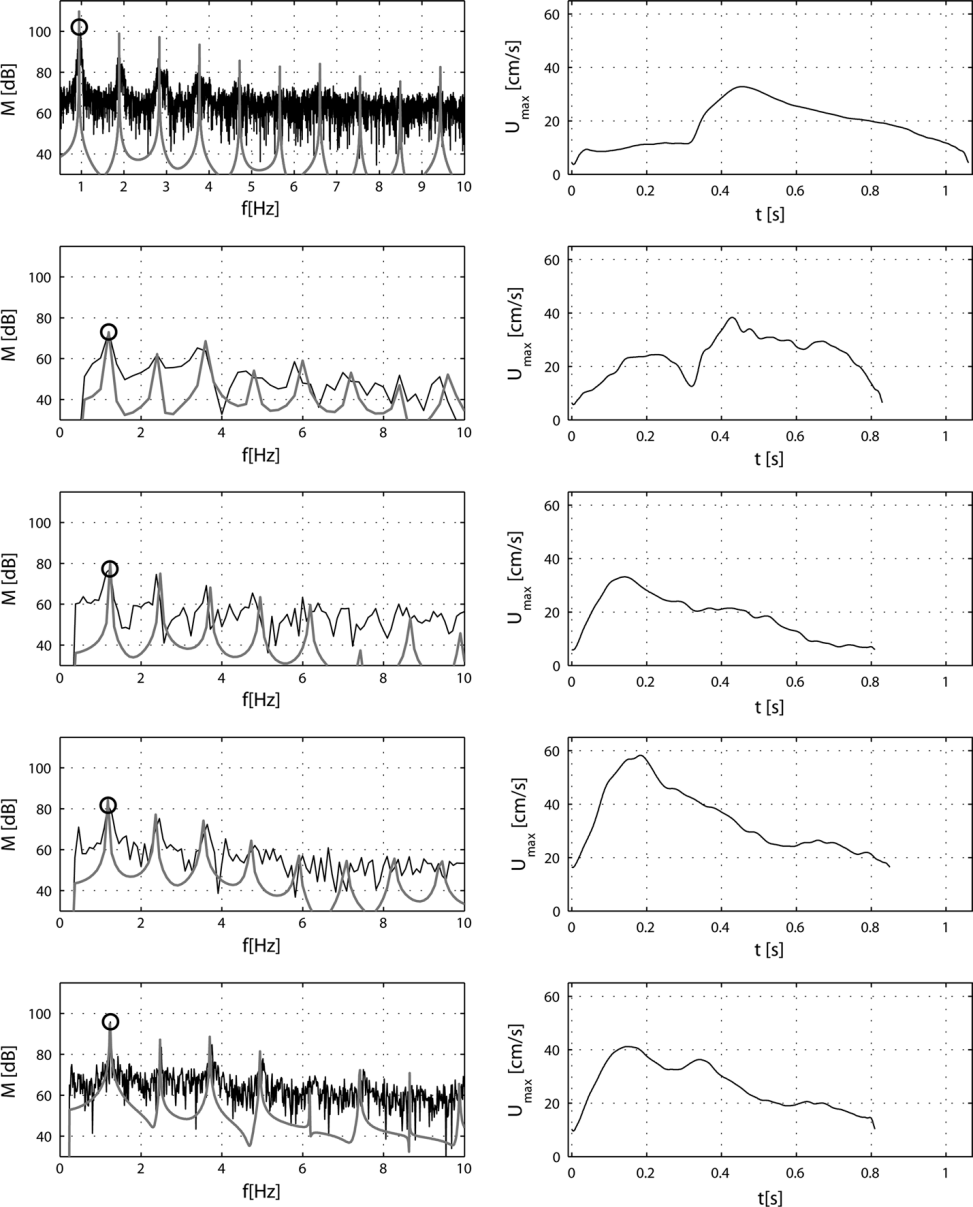
Calculated Fourier spectral analysis and filtered maximum velocities. The Fourier spectral analysis was performed in order to show the filtering effect on the processed signals (*left-hand panel*). In the *left panel*, the analyses are presented for the first 10 harmonics—*black line*: unfiltered signals, *gray line*: signal after filtering;* circle*: fundamental frequency of the heart cycle; the *right-hand panel* presents filtered maximum velocities for all examined patients. The five* rows* from *top* to *bottom* represent patients from 1 to 5.

The experimental data obtained by in vivo measurements in the coronary artery of all patients were recorded for further analysis. The resulting measured physiological data shows variations in the time period and velocity between each time cycle (Fig. 2). The variations indicate that the heartbeats are not constant and there are natural variations between cycles. Moreover, there are perturbations/artefacts in the measured data (not shown). Therefore, the measured data had to be processed in order to remove aberrant data and perturbations as well as to extract a representative signal for the measured velocity. The principle of the filtering strategy is presented in Fig. 2. Firstly, the in vivo data were processed to remove perturbed data probably caused by movements of the patient and/or the measurement sensors. The whole periodical signal was then cut into period signals, in the values, where minimum of a periodic velocity is, marked as circles (Fig. 2a). The cut periods were then filtered based on the following algorithm: The maximum, mean and minimum velocity, as well as the time period of specific cut periods should not vary more than 20% from the average respective value. However, the resulting physiological data still showed noticeable variability in the time period and the velocity between time cycles. This variability is illustrated in Table 1 and Fig. 2b. As shown for the first patient in Fig. 2b, the maximum velocity varied from 23.9 to 41.9 cm/s and the minimum velocity from 0.2 to 7.2 cm/s; the time period varied from 0.93 to 1.19 s. Therefore, to obtain a representative period, an average of all the filtered cycles was constructed, shown as the solid black line in Fig. 2b. The resulting average artery velocity signal was decomposed into a complex Fourier series for further analysis. We used 50 harmonics to obtain good accuracy in the Fourier representation of velocity for each patient. The mean relative error was less than 0.5% for all cases and bellows the standard deviation resulting from the time variability of the measured signals. The minimum, maximum and average results for all patients are given in Table 1. Results of the Fourier decomposition are shown in Fig. 3. The right panel (from top to bottom) illustrates the maximum filtered velocities. The velocity data varied from patient to patient both in intensity and curve shape. The Fourier series spectral analysis was also drawn in order to illustrate the filtering effect on the processed signals (Fig. 3, left-hand panel, from top to bottom). Results show that the filtering effects were different from patient to patient. In patients 1 and 5, the spectral peaks were not altered by filtering. In contrast, the noise that appeared in patients 2, 3 and 4 required a strong filtering to obtain a representative signal. As a result, the number of representative cycles had to be reduced; therefore, for these patients, there is a shift between the spectral peaks.

### Signal analysis and flow models

For the in vivo measurement part of this work, the critical question relates to the diversity of in vivo velocity signals. Despite this variation, a method that allows us to deduce desired additional flow properties such as flow rate, wall shear rate, wall shear stress and wall shear stress time derivative has to be found. Moreover, as mentioned, the main purpose is to find the best method to quantify the flow properties from in vivo and in vitro measurements for further in vitro tests. The chosen method has to be accurate enough to reasonably describe properties of blood flows. Furthermore, it has to be fast enough in order to take as few computer resources as possible and easy to implement in any existing programming language such as C, Matlab^®^ or Labview^®^.

For that purpose, the following elements had to be taken into account. First, as mentioned above, at the first stage, in vitro experiments will be carried out on a straight, rigid and circular tube, seeded with endothelial cells. Moreover, the medium that will be used to fill the in vitro circuit is a Newtonian medium. The flow in most coronary arteries excluding the heart, perturbations in the arteries such as stenosis and big arteries has a laminar character. These physical simplifications of the future in vitro model lead to many mathematical simplifications which allow to select the most widely used method in the blood calculations i.e. Womersley’s solution [18, 19]. This is the analytical solution obtained by integration of the unsteady Navier–Stokes equation in a circular vessel. Womersley’s solution is a valid solution for a Newtonian liquid with a constant viscosity. However, blood viscosity has non-Newtonian character. Therefore, a 2D axisymmetric, non-Newtonian CFD simulation was used to validate the assumption of an approximate Newtonian behavior of blood. Below, briefly, are presented equations that were used to calculate physical quantities and assess the validity of assumptions. For the analytical calculations a script in Matlab^®^ program was written.

In order to check the validity of the first assumption—the laminar behavior of the fluid- the patient’s maximum mean velocity was taken into account in order to find the corresponding Reynolds number using as the reference value the pipe diameter and the mean velocity. A maximum Re number of 442 for patient 4 was found and it validated the assumption that the flow has a laminar behavior for the analyzed vessels.

The second assumption concerns the circularity of the vessel. In the first patient the coronary artery had a slightly elliptical section. The measured diameters of the coronary artery had an average size around 3.48 × 3.30 mm. This could correspond to reality or could be explained by limits in angiography resolution. In fact, we can assume that the section of the coronary artery has a circular geometry and that it will not have a significant influence on the equation solution when the ratio between the small diameter (a) and the big one (b) is higher than 0.9 [19], Eq. 1. In our case, the ratio is 0.948, and therefore we took an approximate circular diameter of 3.387 mm based on the equation. Therefore a circular section was assumed for all patients. Diameters vary depending on patients between 3.387 mm for the first patient until 2.01 mm for the patient 3 (see Table 1).

1 $${\text{d}} = \frac{{2{\text{ab}}}}{{{\text{a}} + {\text{b}}}}$$

where a and b are diameters of ellipse.

The third assumption consists in the approximation of a blood vessel as a rigid pipe. This will use in a first setting of our calculations as the in vitro model will be a rigid tube. The systemic human arteries can be subdivided into two types—muscular and elastic—according to the relative compositions of elastic and muscle tissue in their tunica media as well as their size and the makeup of the internal and external elastic lamina. The larger arteries (>10 mm diameter) are generally elastic and the smaller ones (0.1–10 mm) tend to be muscular. For example large, muscular systemic arteries (e.g., femoral artery) increase in radius by about 10% [12, 20]. Moreover, the elasticity of blood vessels decreases with the size of vessel, age of people and/or due to an illness. In this study, diameters of the examined coronary arteries have dimensions 3.387 and 2.01 mm. According to the division presented above, it places them to the small muscular arteries for which change of the diameter is relatively small. Therefore, the assumed approximation of the vessel as a rigid pipe can be valid for these arteries.

The fourth assumption is about incompressibility of the liquid. In reality, like any other fluid, blood has a small compressibility that can be neglected, in particular when studying the local aspect of the flow. Therefore we assumed an incompressible flow with density which depends on the level of hematocrit.

The last but not least assumption regards the non-Newtonian behavior of the blood. In normal conditions blood is a heterogeneous media principally made of about 55% of liquid plasma and of about 45% of red cells in suspension in the plasma. The concentration of red blood cells modifies the apparent viscosity of blood. Moreover, depending on the level of shear rate, that apparent viscosity changes and leads to the non-Newtonian behavior of blood, with viscosity decreasing as shear rate increases. However for our analytical estimation, we can assume that blood viscosity of a vessel whose diameter is larger than 1 mm is relatively constant, as the values of shear rate on the wall passes into a deformation region (above 100 s^−1^) where the viscosity is relatively constant. Therefore for the first calculations we used the average viscosity from Carreau model in the range of shear rate (0–1,000 s^−1^). The values for all patients are presented in Table 2.

**Table 2:** Comparison of the flow properties (minimum, mean and maximum values) between CFD and Womersley’s solution

	Viscosity μ (Pa s)		Flow rate Q (m^3^/s)		WSS τ (Pa)		WSSTD dτ/dt (Pa/s)	
	CFD	Womersley	CFD	Womersley	CFD	Womersley	CFD	Womersley
Patient 1								
Min	3.11 × 10^−3^	3.42 × 10^−3^	5.64 × 10^−8^	2.62 × 10^−8^	−0.42	−0.41	−52.31	−50.10
Mean	3.42 × 10^−3^	3.42 × 10^−3^	8.62 × 10^−7^	8.14 × 10^−7^	0.78	0.73	0	0
Max	9.56 × 10^−3^	3.42 × 10^−3^	1.62 × 10^−6^	1.54 × 10^−6^	1.51	1.51	75.075	72.22
Patient 2								
Min	2.89 × 10^−3^	3.09 × 10^−3^	8.86 × 10^−8^	7.45 × 10^−8^	−0.27	−0.28	−63.41	−53.35
Mean	3.09 × 10^−3^	3.09 × 10^−3^	6.08 × 10^−7^	5.86 × 10^−7^	1.19	1.15	0	0
Max	5.12 × 10^−3^	3.09 × 10^−3^	1.04 × 10^−6^	1.00 × 10^−6^	2.15	2.17	81.50	83.73
Patient 3								
Min	3.62 × 10^−3^	3.85 × 10^−3^	9.32 × 10^−8^	8.62 × 10^−8^	0.42	0.31	−25.90	−23.53
Mean	3.85 × 10^−3^	3.85 × 10^−3^	3.02 × 10^−7^	2.92 × 10^−7^	1.47	1.41	0	0
Max	4.53 × 10^−3^	3.85 × 10^−3^	5.43 × 10^−7^	5.29 × 10^−7^	2.58	2.59	51.96	51.88
Patient 4								
Min	3.00 × 10^−3^	3.11 × 10^−3^	3.19 × 10^−7^	3.03 × 10^−7^	0.73	0.83	−24.91	−25.75
Mean	3.11 × 10^−3^	3.11 × 10^−3^	6.88 × 10^−7^	6.67 × 10^−7^	1.89	1.98	0	0
Max	3.33 × 10^−3^	3.11 × 10^−3^	1.20 × 10^−6^	1.15 × 10^−6^	3.35	3.43	49.30	48.93
Patient 5								
Min	3.55 × 10^−3^	3.69 × 10^−3^	1.52 × 10^−7^	1.44 × 10^−7^	0.22	0.14	−81.80	−74.18
Mean	3.69 × 10^−3^	3.69 × 10^−3^	4.89 × 10^−7^	4.74 × 10^−7^	1.89	1.82	0	0
Max	5.31 × 10^−3^	3.69 × 10^−3^	7.64 × 10^−7^	7.46 × 10^−7^	2.91	2.90	89.56	83.34

Based on the assumptions described above, the flow momentum equation for direction z (flow direction) in a cylindrical system becomes:

2 $$\frac{{\partial {\text{U}}_{\text{z}} }}{{\partial {\text{t}}}} = - \frac{1}{\rho }\frac{{\partial {\text{P}}}}{{\partial {\text{z}}}} + \frac{\mu }{\rho }\frac{{\partial^{2} {\text{U}}_{\text{z}} }}{{\partial {\text{r}}^{2} }} + \frac{\mu }{\rho }\frac{1}{\text{r}}\frac{{\partial {\text{U}}_{\text{z}} }}{{\partial {\text{r}}}}$$

where; ${\text{P}} = {\text{P}}({\text{z}},{\text{t}})$ is the pressure, ${\text{U}}_{\text{z}} = {\text{U}}_{\text{z}} ({\text{r}},{\text{t}})$ is the axial velocity along z axis and assuming no flow in direction θ and r.

Womersley’s solution obtained by the analytical integration of Eq. 2 is based on Fourier series in time and Bessel’s function in space. The final equations used to calculate blood properties in a Matlab script are therefore as presented below.

The peak velocity, at the centre (r = 0) is given by Fourier series:

3 $${\text{U}}({\text{t}},{\text{r}} = 0) = \mathop \sum \limits_{{{\text{n}} = - \infty }}^{\infty } {\text{C}}_{\text{n}} {\text{e}}^{{{\text{i}}\omega {\text{nt}}}}$$

The U(r,t) velocity profile is given by:

4 $${\text{U}}\left( {\text{r,t}} \right) = \mathop \sum \limits_{ - \infty }^{\infty } \frac{{{\text{G}}_{\text{n}} {\text{iR}}^{ 2} }}{{\mu \varOmega_{\text{n}}^{2} }}\left( {1 - \frac{{J_{0} \left( {\zeta_{\text{n}} } \right)}}{{J_{0} \left( {\varLambda_{\text{n}} } \right)}}} \right)e^{{{\text{i}}\omega {\text{nt}}}}$$

where:

5 $$\varOmega_{\text{n}} = \sqrt {\frac{{{\text{n}}\omega \rho }}{\mu }} {\text{R}}$$

6 $$\varLambda_{\text{n}} = {\text{i}}^{3/2} \varOmega_{\text{n}}$$

7 $$\zeta_{\text{n}} = \varLambda_{\text{n}} \frac{\text{r}}{\text{R}}$$

The pressure gradient coefficient G_n_ can be calculated based on the Eqs. (3, 4) for a peak velocity at r = 0. For any nth harmonic of the Fourier series, one can write:

8 $$C_{\text{n}} (r = 0) = \frac{{G_{\text{n}} iR^{2} }}{{\mu \varOmega_{\text{n}}^{ 2} }}\left( {1 - \frac{{J_{0} \left( 0 \right)}}{{J_{0} \left( {\varLambda_{\text{n}} } \right)}}} \right)$$

Therefore,

9 $${\text{G}}_{\text{n}} = \frac{{{\text{C}}_{\text{n}} \mu \varOmega_{n}^{2} }}{{{\text{iR}}^{2} \left( {1 - \frac{{{\text{J}}_{0} \left( 0 \right)}}{{{\text{J}}_{0} \left( {\varLambda_{\text{n}} } \right)}}} \right)}}$$

The pressure gradient along the flow axis z is expressed by:

10 $$\frac{\text{dP}}{\text{dz}}(t) = \mathop \sum \limits_{{{\text{n}} = - \infty }}^{\infty } G_{n} e^{{{\text{i}}\omega {\text{nt}}}}$$

The instantaneous flow rate is given by:

11 $$Q\left( {\text{t}} \right) = \mathop \sum \limits_{{{\text{n}} = - \infty }}^{\infty } \frac{{G_{\text{n}} iR^{4} }}{{\mu \varOmega_{\text{n}}^{2} }}\left( {1 - \frac{{2J_{1} \left( {\varLambda_{\text{n}} } \right)}}{{\varLambda_{\text{n}} J_{ 0} \left( {\varLambda_{\text{n}} } \right)}}} \right)e^{{{\text{i}}\omega {\text{nt}}}}$$

The wall shear stress (WSS) is given by:

12 $$\tau \left( {\text{t}} \right) = - \mathop \sum \limits_{{{\text{n}} = - \infty }}^{\infty } \frac{{G_{\text{n}} R}}{{\varLambda_{\text{n}} }}\left( {\frac{{J_{1} \left( {\varLambda_{\text{n}} } \right)}}{{J_{0} \left( {\varLambda_{\text{n}} } \right)}}} \right)e^{{{\text{i}}\omega {\text{nt}}}}$$

For a Newtonian fluid, the wall shear rate $\dot{\gamma }$ is linked to the shear stress by:

13 $$\tau \left( {\text{t}} \right) = - \mu \dot{\gamma }$$

And the wall shear stress time derivative (WSSTD) is given by:

14 $${\text{WSSTD}} = \frac{d\tau }{dt}$$

Although none of the assumptions quoted above are 100% true for blood flows, most of them are a fair approximation of reality. Moreover, to assess the effect of the non-Newtonian character of blood, a CFD computation was performed on COMSOL^®^ Multiphysics software. These CFD calculations are based on the unsteady Navier–Stokes equations that for incompressible and non-Newtonian fluid lead to:

15 $$\vec{\nabla } \cdot {\vec{\text{u}}} = 0 {\quad\text{mass equation}}$$

16 $$\rho \frac{{\partial \vec{u}}}{\partial t} + \rho \left( {\vec{u} \cdot \vec{\nabla }} \right)\vec{u} = - \vec{\nabla }p + \overrightarrow {\nabla } \cdot \left( {\mu \left( {\overrightarrow {\nabla \otimes } \vec{u} + \left( {\overrightarrow {\nabla \otimes } \vec{u}} \right)^{T} } \right)} \right) + \rho \vec{f} {\quad\text{momentum equation}}$$

where, f is external volume force, $\mu$ is dynamic viscosity that depends on shear rate $\dot{\gamma }$ with $\dot{\gamma } = \vec{\nabla }\overrightarrow {{ \otimes {\text{u}}}} + \left( {\overrightarrow {\nabla \otimes } {\vec{\text{u}}}} \right)^{\text{T}}$.

In order to describe non-Newtonian behavior of blood a mathematical model is needed. As mentioned above, the Carreau model was used to model blood viscosity in function of shear rate and in addition in function of the hematocrit level based on the experimental viscosity measurements [17]. It is four parameters model where these parameters are expressed in function of the hematocrit level (Table 3) as follows [17].

**Table 3:** Carreau model coefficients [17] and hematocrit levels required for CFD calculations

Model	Patient 1	Patient 2	Patient 3	Patient 4	Patient 5
μ_0_ (Pa s)	2.30 × 10^−2^	2.17 × 10^−2^	2.77 × 10^−2^	2.34 × 10^−2^	2.74 × 10^−2^
μ∞ (Pa s)	2.73 × 10^−3^	2.61 × 10^−3^	3.30 × 10^−3^	2.77 × 10^−3^	3.25 × 10^−3^
λ (s)	1.39	1.48	1.10	1.36	1.12
p (-)	0.39	0.40	0.35	0.39	0.36
Hematocrit (%)	38.0	36.6	42.7	38.4	42.4

17 $$\mu (\dot{\gamma }, {\text{f}(\text{Hct}})) = \mu_{\infty }^{\text{f}(\text{Htc})} + \left( {\mu_{0}^{\text{f}(\text{Htc})} - \mu_{\infty }^{\text{f}(\text{Htc})} } \right)\left( {1 + \left( {\lambda^{\text{f}(\text{Htc})} \dot{\gamma }} \right)^{2} } \right)^{{\frac{{p^{\text{f}(\text{Htc})} - 1}}{2}}}$$

Assuming that vessels are circular a 2D axis symmetric space dimension was used. A quadrangle element for mesh was chosen. However, in order to resolve the boundary layers on solid boundaries an anisotropic boundary layer mesh was added. This type of mesh stretches the mesh cells so that the velocity gradient close to the wall is captured. The length of the cylindrical domain, representing as simplified vessel, was chosen in order to ensure a good compromise between CPU/memory cost and simulation accuracy. The length had to be as short as possible to reduce the calculation cost but long enough to ensure that the flow will be fully developed. The minimum length required to have a fully developed a steady laminar flow could be calculated with a semi-empirical law [21]:

18 $$\frac{{{\text{L}}_{\text{e}} }}{\text{D}} \approx 0.0575{\text{Re}}$$

Based on the Eq. (18), the length of the pipe for all examined here diameters was set at 0.05 m and the measurement point for the comparison of all data was located at 0.025 m. The 2D axial symmetry model requires four boundary conditions:

1. A symmetry constraint with an axial symmetry condition at r = 0.
2. An inlet boundary condition with normal inflow velocity. Starting with a mean velocity equals to half of the peak velocity from the in vivo data, an iterative adjustment is performed on that inlet mean velocity in order to fit the unsteady in vivo measured peak velocity as follows:
  19 $${\text{U}}_{\text{mean}}^{{{\text{n}} + 1}} = \frac{{{\text{U}}_{ \rm{max} }^{{{\text{n}} + 1}} }}{{{\text{U}}_{ \rm{max} }^{\text{mes}} }} \cdot {\text{U}}_{\text{mean}}^{\text{n}}$$
  where, ${\text{U}}_{\text{mean}}^{{{\text{n}} + 1}}$ is a new mean velocity for an inlet condition, ${\text{U}}_{\text{mean}}^{\text{n}}$ is an old mean velocity for an inlet condition, ${\text{U}}_{ \rm{max} }^{{{\text{n}} + 1}}$ is a new calculated maximum velocity, ${\text{U}}_{ \rm{max} }^{\text{mes}}$ is a measured maximum velocity.
3. An outlet boundary condition with an effective pressure (relative pressure with a reference pressure level set at 0 Pa.
4. No slip wall condition.

The iterative steps were repeated until the local maximum velocity of in vivo velocity vs. CFD velocity reached relative error a value less than 1%.

### Design of the in vitro test bench

**Fig. 4:**
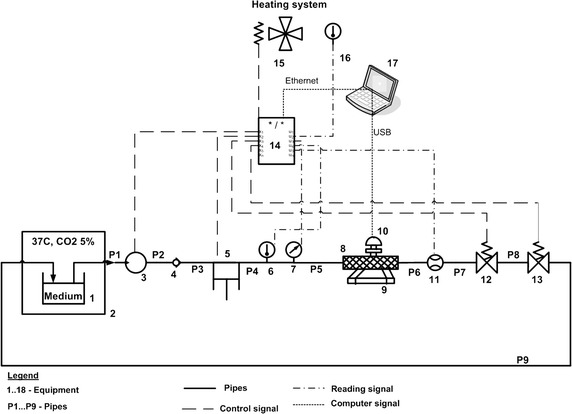
The in vitro test bench. The different parts of the in vitro test bench are depicted. *1* reservoir, *2* incubator, *3* centrifugal pump, *4* one–way valve, *5* custom designed piston pump, *6* thermocouple, *7* pressure sensor, *8* test section, *9* microscope, *10* CCD camera, *11* flow meter, *12, 13* solenoid proportional valves, *14* AC/DC controller, *15* heating system, *16* thermocouple, *17* PC class computer/notebook/touch screen computer.

The general outline of the test bench is presented in Fig. 4. The circuit starts in an incubator (2, Revco Ultima II RMI3000 Thermo Scientific) where a reservoir (1) filled with a medium DMEM (Dulbecco’s Modified Eagle’s Medium) with 1 g Glucose, 25 mM Hepes and supplemented with: 10% FBS (Foetal Bovine Serum), 1% l-Glutamine, 1% Penicillin + Streptomycin, 2% HAT (Hypoxanthine Aminoptein Thymidine) and 1% NEAA (Non-essential amino acid). In order to set the desired viscosity level, a Dextran complex is used. The reservoir is placed in the incubator in order to maintain physiological conditions (pH level at 7.45). This is done by keeping the gas balance at the proper level i.e. CO_2_ 5% and air 95%. The mean flow rate is ensured by a centrifugal pump (3, BPX-50 Biopump Medrotronic) which is specifically designed to pump blood. A one-way valve (4, W96029 Clapet Kynar) and a customised piston pump (5, PS01-23 × 80F-HP-R, Linmot) are connected to a linear motor that controls oscillations over the mean flow rate. The temperature level (37°C) of the medium is read on the thermocouple (6, PR-11-2-100-M60-100-E-CLA, Omega). Just before the test section (8), a pressure sensor (7, CTE8N01GY0N, Sensortechnics) measures the pressure inside the system. The test section is on-line with a microscope (9, DMIL, Leica) connected to a digital camera (10, Infinity 2-5C, Lumenera). The camera is able to take pictures of ECs seeded in the test section while the system is working. The flow rate is measured by an electromagnetic flow meter (11, MM-04-XX-VD-SS-XX-0000-22-1A-1F-00-2R-00-SK, Minimag). The last two pieces of equipment inserted in the circuit are solenoid proportional valves (12, 13, type 2833, Bürkert). All devices are connected by flexible tubing (Tygon B-44-4X, Masterflex). To ensure sterility and mobility, the system is enclosed in a polycarbonate transparent box. Temperature of the medium is controlled by a heater incorporated in the system (15, Cirrus 40 PTC fan heater, DBK). The whole package is controlled by National Instruments real-time CompactRIO^®^ system (NI9024) controller under LabVIEW™ environment. The control strategy is based on the closed loop with Internal Model Control and supported PID controllers in order to allow a feedback to all measured changes of the environment such as temperature, viscosity, flow or pressure.

### Control strategy of the in vitro test bench

**Fig. 5:**
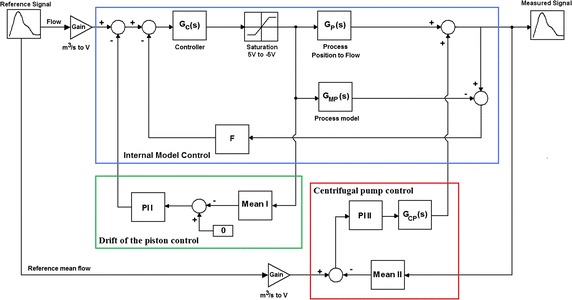
The concept of the control system. The main control strategy consists in three parts. The internal model control (marked as a *blue square*) is responsible to control the position of the piston pump. In order to reduce noises from measured flow signal as well to avoid drift of the piston a filter (marked F) and an additional loop which is based on a proportional-integral controller and a function Mean I were incorporated (marked as a *green square*). To control the mean flow rate a proportional-integral controller and a function Mean II were added (marked as a *red square*).

The principle of the control loop of the flow pulsatile rate is presented in Fig. 5. The reference signal is divided into two signals: the mean flow and the pulsatile fluctuations of the flow. From that division, we have two main controls. In the first control (Centrifugal pump in Fig. 5 as a red square), the mean flow is controlled by the PI controller number II and the function “mean II”. The function “mean II” is responsible for calculating the mean flow at each cycle. It adds every point of the measured flow cycle and divides the latter by the number of the measured samples for this cycle. Then the signal is sent as a measured signal to the adder in order to compare with the mean reference value. After that, the controller PI II sets the rotation of the centrifugal pump in order to get the desired mean flow rate [the transfer function G_CP_(s)]. Thanks to the function “mean II” the control loop is less sensitive to disturbances on the mean flow, which helps to achieve the stability of the whole system. The second control loop is responsible for controlling the shape of the pulsatile flow. This loop is based on the Internal Model Control (Internal Model Control in Fig. 5 as a blue square) and is additional to the PI controller. Basically, the reference signal is compared with a measured signal and is sent to the internal controller G_C_(s). After that, the controlled signal goes through a saturation block which sets the movement limits of the linear motor. The signal is then sent to the controller of the linear motor and drives the movements. Later the flow is read by the electromagnetic flowmeter that is presented in Fig. 5 as a transfer function between the position of the linear motor and flowmeter G_P_(s) and in addition is read by model process G_MP_(s) in order to predict the position of the linear motor. The signal then passes by a filter F and is compared with the actual position of the body. In addition, in order to prevent the linear motor from drifting, we incorporated an additional loop (Drift of the piston in Fig. 5 as a green square). This loop calculates a mean position for every cycle separately and compares it with the mean position previously set. If there is any drift, the PI controller number II modifies the reference signal of the flow. The purpose of the system is to be as close as possible to reality. In the real human body, the condition that keeps the cells alive is temperature, which must be kept between 36 and 38°C. From a fluid-mechanics point of view the constant temperature helps to maintain certain conditions of the liquid such as viscosity. Viscosity is an important variable in the investigated system as it plays a role on the response of the system and is used to calculate the shear stress that acts on the vascular cells. Therefore, the additional heating system had to be incorporated in order to control the temperature of the medium. The heat is controlled by a PTC heater with a fan (Fig. 4, marked as 15 and 16). The control strategy is based on the temperature measured by the liquid thermocouple and the air heater with the fan. Basically, the thermocouple measures the apparent temperature of the liquid and compares it with the desired one. If there is a difference, the control loop sends a signal to the relay (switch) that opens or closes the electrical circuit and directly powers the heater and air inside the box is heated.

## Results

**Fig. 6:**
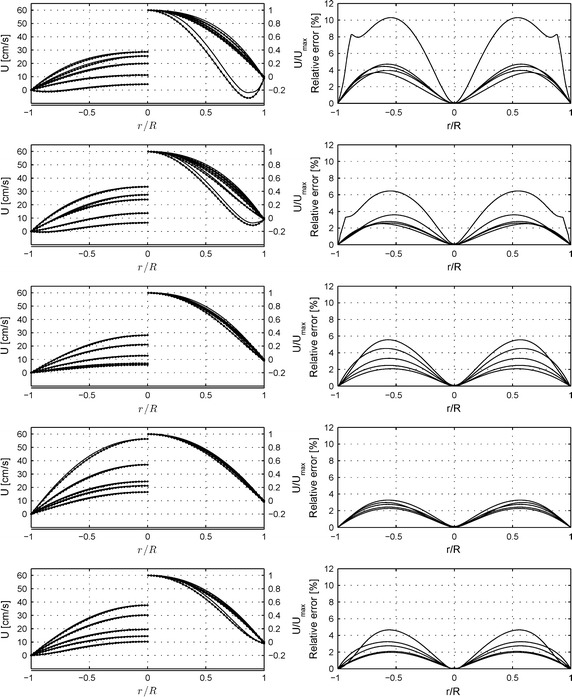
Comparison between Womersley’s solution and non-Newtonian 2D axisymmetric CFD simulations: velocity profiles and relative errors. In order to validate Newtonian assumption of the Womersly’s solution velocity profiles were drawn (*1st column*) at time steps t = 0 s, 0.2 s, 0.4 s, 0.6 s and 0.8 s (not presented). *Graphs* present two solutions: The local velocity for each method (left part of the graph); The dimensionless velocity profiles i.e. the local velocity divided by maximum velocity (*right part* of graph)—*solid lines* CFD simulations,* solid lines with bullets* Womersley’s solution. The* 2nd column* presents relative errors between both methods for velocity profiles.* Rows* from *up* to *down* present patients from 1 to 5.

**Fig. 7:**
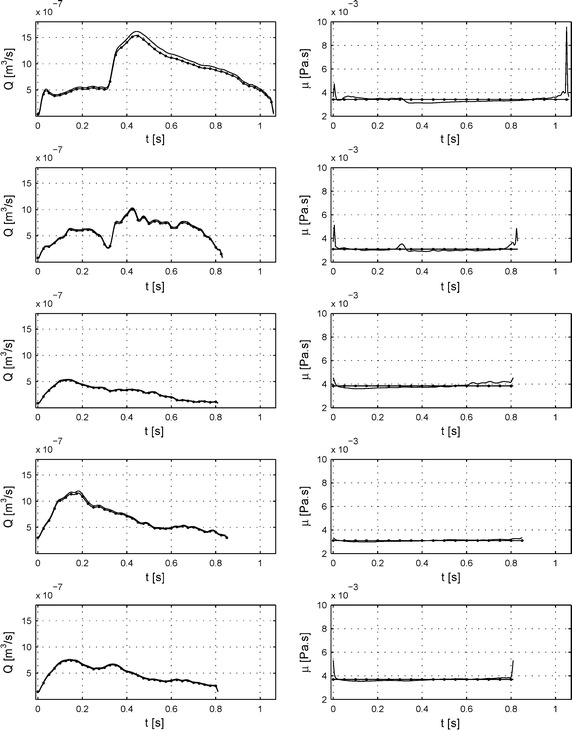
Comparison between Womersley’s solution and non-Newtonian 2D axisymmetric CFD simulations: flow rate and viscosities. One of the most important physical properties is flow rate that is presented in the* 1st column *for one period cycle. The* 2nd column *presents changes of viscosities during one cycle period—*solid lines* CFD simulations based on Carreau model,* solid lines with bullets *Womersley’s solution based on constant viscosity.* Rows* from *up* to *down* present patients from 1 to 5.

**Fig. 8:**
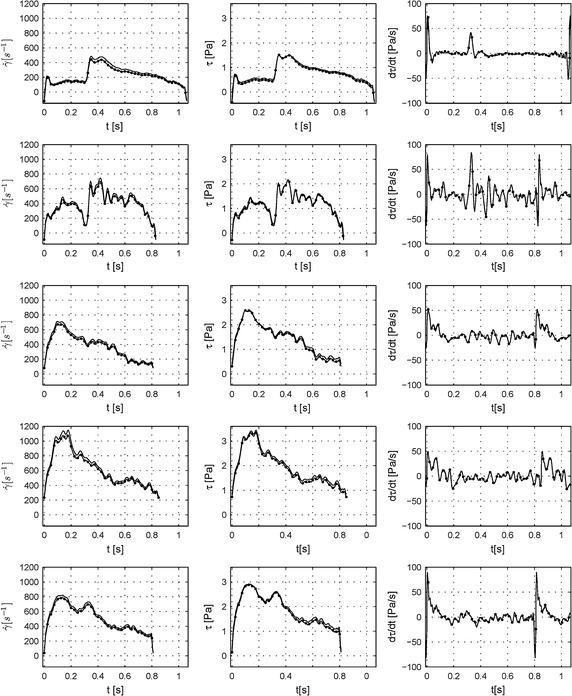
Comparison between Womersley’s solution and non-Newtonian 2D axisymmetric CFD simulations: wall shear rate, wall shear stress and wall shear stress time derivative. Vascular endothelial cells respond is strongly connected to the hemodynamic properties such as wall shear rate (*1st column*), wall shear stress (*2nd column*) and wall shear stress time derivative (*3rd column*)—*solid lines* CFD simulations,* solid lines with bullets* Womersley’s solution. *Rows* from* up to down* present patients from 1 to 5. All graphs are drawn for one period cycle.

In order to extract additional physical quantities such as flow, wall shear stress and wall shear stress time derivative from in vivo measurements, they had to be post-processed. In this work we aimed to compare the two methods to calculate the parameters of interest: CFD calculation and Wormesley’s solution. The results of these comparisons are presented in Figs. 6, 7, 8. After the in vivo measurements using the ComboWire^®^ (see “Methods”), the velocity profiles inside coronary arteries were calculated (Fig. 6, 1st column). The calculated values are shown for time steps 0, 0.2, 0.4, 0.6 and 0.8 s. The maximum velocity was set on the basis of in vivo measurements. It is therefore logical to detect no difference at the centre r = 0 (Fig. 6, 2nd column). The main observations are the following: the relative error increases while velocity decreases. The largest error was found for patient 1 (maximum 10%) and the smallest for patient 4 (maximum 3.2%). Womersley’s solution underestimates values as compared to CFD. For example, compare solid line (CFD calculation) and solid line with bullets (Womersley’s solution) for patient 1. Velocity profiles become slightly flat in the centre of the arteries compared to typical Newtonian solution and it is well presented for patient 1 and 2. In some cases, patients present negative values next to the wall at time step 0 s (patient 1 and 2).

The differences in the velocity profiles could be related to the different methodologies used to calculate the viscosity in the two methods, as in the Womersley’s solution an average viscosity was taken, while for CFD calculation the Carreau model was used with parameters presented in Table 3 [17]. Calculated viscosities are shown in Fig. 7, 2nd column. The main observations are: Cycles begin and end with higher values which can show as abrupt peaks (as seen in patient 1 and 2 for example Fig. 7, 2nd column, graphs 1 and 2 solid lines). The fluctuations over the average viscosity (solid line with bullets) can be important (as seen for first patient Fig. 7, 2nd column, graph 1). Furthermore, blood viscosity strongly depends on the applied shear rate and varies a lot between shear rate 0–100 s^−1^ [12]. The analysis of shear rate level for all patients (Fig. 8, column 1st), shows that during a fraction of the pulsatile period the shear rate is in that range i.e. between 0 and 100 s^−1^. Even more, the shear rate can also drop under zero value and achieve negative values. These patients display the most variable viscosities and the largest differences between the investigated methods (again in patients 1 and 2).

The flow rate is the physical property that set the conditions for system control. Therefore we calculated flow rates for all patients as shown in Fig. 7 and 1st column. Although the shape of the pulsatile flow rate is similar, the flow rate predicted with CFD is higher than the one obtained with the analytical Womersley’s solution. Nevertheless, a similar shape of flow pattern was deduced from calculations by both methods: For all patients, a similar flow rate peak for the systolic part and a slowly decreasing flow rate during the diastolic part were observed. We can notice that the flow pattern as well as the regularities or irregularities in the flow varies strongly form one patient to the other. The higher flow was observed for patient 1 and the lowest for patient 3 (Fig. 7, 1st column, graphs 1 and 3). Besides, the second patient presents a very irregular flow rate (Fig. 7. 1st column, graph 2). That strong physiological variability is a key and challenging issue for the control system of an in vitro system that should mimic accurately the in vivo behaviour.

Finally we also calculated physiological properties that we would like investigated the most i.e. wall shear stress (WSS) and wall shear stress time derivative (WSSTD), as shown in Fig. 8, 2nd and 3rd columns. The differences between the two calculation methods are not significant. The curve obtained using analytical solution follows the shape of CFD calculations for all patients and achieves almost the same maximum and minimum values for WSS as well as for WSSTD. As can be noticed, for patient 1 and 2, the velocity profile presents some reverse flow near the wall during a part of the pulsatile cycle (Fig. 6, 1st column). It is well presented for patients 1 and 2 for time step of 0 s. This is also marked by the fact that the WSS becomes negative (changes direction) during these phases. The WSS is the highest for patient 4 and the lowest for patient 1. All WSSTD also presents a peak at the beginning and end of the cycle. Between these two peaks oscillations can vary from patient to patient there are more oscillations for patients 2–4 comparing to patients 1 and 5. Here again, Womersley’s solution slightly underestimated values as compared to CFD values. The lowest and highest values can be found for patient 5. Table 2 summarizes minimum, mean and maximum values for all calculated physical quantities.

To validate the in vitro test bench conception a comparison between post-processed in vivo and in vitro measurements was performed. Thus the velocity measured in vivo using the combo wire as well as flow rate measured in vitro by the flow meter were processed for further analysis. The comparison was based on the same methodology i.e. the Womersley’s solution either set as a desired signal or used to calculate desired flow-generated quantities such as WSS or WSSTD.

**Fig. 9:**
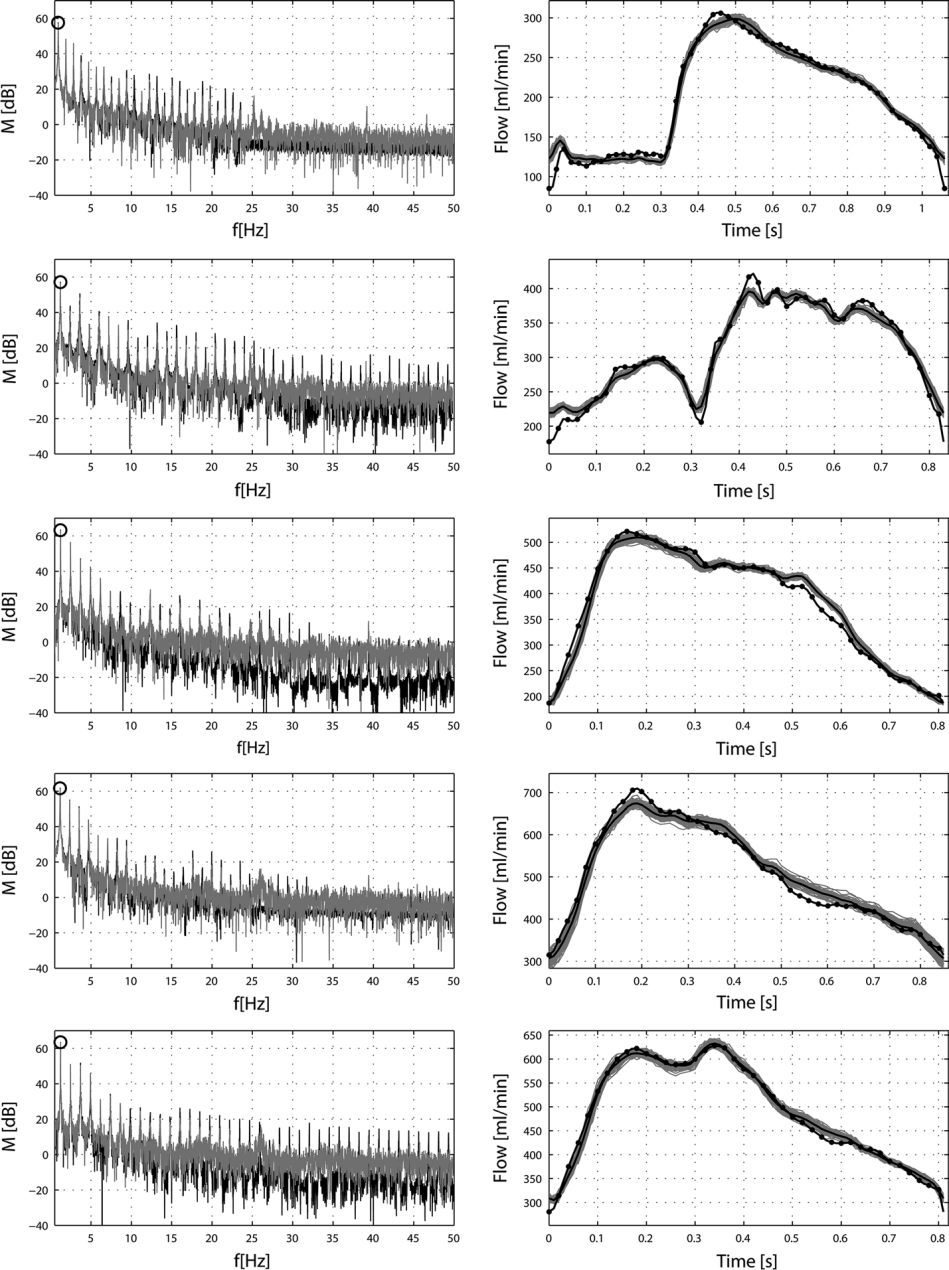
Comparison between flow rates calculated by the Womersley’s solution in in vivo blood vessels and in the in vitro test bench. The Fourier spectral analysis was calculated in order to assess the differences in reproduced frequencies between Womersley’s solution (*black line*) and ones obtained in vitro (*grey line*) (*left-hand panel*). On that graph the circle point out the fundamental frequency of the heart cycle. The *right panel* is presented flow rate obtained using the in vitro test bench. The *solid black line* with points presents desired signal, the *solid grey line* presents measured cycle, *solid black line* presents average of 60 measured cycles.* Rows* from *top* to *bottom* show patients from 1 to 5.

At first, we compared the flow rate that constitutes the physical quantity set as parameter for controlling the system. The results obtained for five patients are presented in Fig. 9, right panel and Table 4. To assay the capacity of the system, the signals were recorded during a time period of 60 s and then post-processed by decomposing the whole signal in cycles (Fig. 9, right panel grey lines). The average signal was then calculated by adding individual cycles and set as a reference signal (Fig. 9, right panel black line). All further calculations were made based on this average signal. It is worth emphasis that the signal can be precisely followed with a reproducibility having variability below 7% for all investigated patients. The measured flows fitted well the limits described hereinabove. Moreover, the achieved flows accurately reproduce the reference in vivo signals with a mean relative error below 5% for all investigated signals. However, the control strategy still has difficulties to adapt to rapid changes (peaks) of the flow and biggest discrepancies were noticed at these points. To visualise differences, the Fourier spectral analysis were calculated (Fig. 9, left-hand panel).

**Table 4:** Comparison of the flow properties (minimum, mean and maximum values) as calculated by non Newtonian unsteady CFD simulations and Womersley’s solution

	Viscosity μ (Pa s)	Flow rate Q (ml/min)		WSS τ (Pa)		WSSTD dτ/dt (Pa/s)	
	Womersley	Womersley	In vitro	Womersley	In vitro	Womersley	In vitro
Patient 1							
Min	3.42 × 10^−3^	85.2	118.2	−0.16	0.22	−22.01	−13.88
Mean	3.42 × 10^−3^	197.8	198.4	0.73	0.74	0	0
Max	3.42 × 10^−3^	306.5	298.8	1.50	1.50	37.14	25.95
Patient 2							
Min	3.09 × 10^−3^	177.7	219.6	−0.30	0.37	−64.40	−19.86
Mean	3.09 × 10^−3^	310.4	310.9	1.14	1.15	0	0
Max	3.09 × 10^−3^	421.5	395.7	2.16	1.88	75.13	36.09
Patient 3							
Min	3.85 × 10^−3^	186.7	187.1	0.33	0.42	−25.13	−14.05
Mean	3.85 × 10^−3^	380.7	380.9	1.41	1.41	0	0
Max	3.85 × 10^−3^	521.1	509.6	2.56	2.69	43.75	27.55
Patient 4							
Min	3.11 × 10^−3^	315.3	307.2	0.77	0.77	−21.09	−13.83
Mean	3.11 × 10^−3^	508.6	508.4	1.88	1.88	0	0
Max	3.11 × 10^−3^	709.9	674.5	3.27	3.09	45.28	32.04
Patient 5							
Min	3.69 × 10^−3^	280.6	305.2	0.13	0.71	−84.83	−13.16
Mean	3.69 × 10^−3^	488.9	489.7	1.80	1.81	0	0
Max	3.69 × 10^−3^	627.8	632.6	2.87	2.89	87.48	28.21

**Fig. 10:**
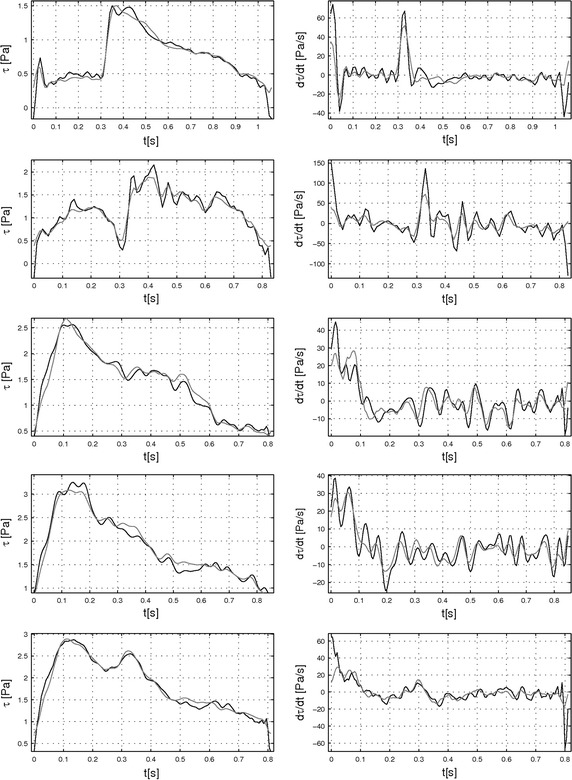
Comparison between wall shear stress and wall shear stress time derivative calculated by the Womersley’s solution in in vivo blood vessels and in the in vitro test bench. Hemodynamic properties, such as the wall shear stress (*left panel*) and wall shear stress time derivative (*right panel*) were calculated from Womersley simulations of in vivo data (*solid black lines*) and from reproduction by our in vitro test bench (*solid grey lines*) and plotted against time. *Rows* from *top* to *bottom* refer to patients from 1 to 5. All graphs are drawn for one period cycle.

Based on the experimental in vitro data as well as in vivo data, two important physiological quantities i.e. wall shear stress (WSS) and wall shear stress time derivative (WSSTD) were also derived and compared. They are shown in Fig. 10. The differences observed between the in vivo and in vitro averaged data were correlated to the flow values measured and presented in the previously paragraph. Like for the flow, the main shape of the WSS as well as the WSSTD (grey line) generated in vitro matched well the reference signal generated from in vivo *data* (black line). However, as mentioned for the flow results, the calculated physical quantities for in vitro data are mostly underestimated compared to calculated in vivo data. Again, periods of rapid changes (peaks) of the WSS/WSSTD were associated to the largest calculated discrepancies. Moreover, due to the difficulties encountered in mimicking the fast change of the WSS/WSSTD it was not possible to reconstruct accurately enough negative values of the WSS/WSSTD.

## Discussion

In vivo pulsatile blood velocity data were successfully obtained for five patients using a Doppler flow ComboWire^®^ (Volcano Corp.), although there were some difficulties to obtain the Doppler flow signal. For example, placing a wire in a suitable position, i.e., as centrally as possible in order to get sufficient signal intensity took several minutes, an inconvenience probably related to the difficulty in choosing the best coronary artery/place to perform an accurate measurement i.e. to measure maximum velocity avoiding any artifact and noises. Patients were selected from those needing coronagraphy according to the ethical board’s policy, and we were limited to the use of sites where coronagraphy had to be performed. Within these restrictions, our goal was to find healthy vessels without stenosis and as straight as possible in order to obtain proper measurements. Nevertheless, some measurements had to be performed close to branches or curves. These practical problems add to the inherent variability between successive human heart beats, perturbed signals due to movements of the patient, etc. Hence, the data obtained were often noisy and additional filtering was needed.

After filtering, the five different in vivo signal measurements were analyzed and post-processed. Results showed that the flow pattern varied widely among patients and confirmed the reported variability of pulsatile flow waveforms observed in the arteries (see, e.g., Mills et al. [22], McDonald [23]). The differences among patients in the magnitude of the velocities as well as in the shape of the curve could be due to intrinsic differences between patients and/or measurement positions.

The main goal was to perform experiments with the simplest settings. First in vitro tests involved straight rigid pipes containing a medium with Newtonian properties. However, the necessary velocity measurements had to be made with blood, which has non-Newtonian behavior. In this study, we extracted detailed information on the physical flow quantities, such as pulsatile flow, WSS and WSSTD from in vivo measurements using the Wormesley’s solution (usually applied to a Newtonian behavior). Our subsequently comparison with numerical non-Newtonian, based on the Carreau model, CFD calculations indeed validated the Womersley’s solution for five patients tested. The mean relative errors for the flow rate vary between 3.2 and 6.3%. As presented, both techniques give rise to similar results with a difference growing for smaller flow rates (Figs. 6, 7, 8). Thus, the Womersley’s solution is a fast method that can be used to provide satisfying approximations especially for the higher flow rates where shear rate is higher than 100 s^−1^ and where the non-Newtonian behavior becomes negligible. For some cases, where more accurate and detailed analysis is required, is should nevertheless be recommended to use CFD techniques.

Based on the previous results and the Womersley’s solution, the comparison with experimental in vitro data was performed. The results in Table 4 suggest that the concept behind the whole control system is valid. The mean relative error is below 5% for all investigated signals. In addition, we have to bear in mind that we are dealing with very fast control loops. LabVIEW™ executes one cycle for all the input/output signals in a time period of 5 ms (200 Hz). It is much quicker than in most industrial controls of mechanical systems. Also, the system is highly non-linear and depends on external conditions such as medium composition that can vary a lot depending on supplements or serum. Other sources of variability include properties of the equipment such as, for example the diaphragm age, external temperature and so on.

The results obtained for all examined patients with the real-time system are satisfactory. However, the system experienced difficulties to follow rapid flow changes. Therefore, when fast WSS and WSSTD acceleration/deceleration appeared, a large error was generated. This results from the foundations behind the WSS calculation method. It indeed involves the use of derivatives which amplifies the noise particularly at higher frequencies. As the system struggles to reproduce fast changes, the calculated WSS values between the reference flow and measured flow may therefore display significant discrepancies, reflected in the calculated relative error. The same principle may be applied to WSSTD, whose values are function of a second derivative of the flow. Therefore the relative error becomes even higher.

As mentioned earlier, several studies have correlated shear stress perturbations with EC behavior. Recently, other studies have correlated WSSTD with the development of the atherogenic phenotype [8], with for example, WSSTD-induced EC proliferation, upregulation of platelet-derived growth factor (PDGF-A) and monocyte chemoattractant protein-1(MCP-1) and enhanced monocyte binding [8]. Investigations have also demonstrated effects of WSSTD and temporal gradient in shear on EC remodeling [8, 24]. However, the WSSTD signal in these studies was deduced as the result of ramp, step, sinusoidal or impulse laminar flow. In this paper, we calculated WSSTD (Figs. 8, 10, right-hand panel) based on real in vivo and in vitro measurements. Clearly WSSTD signals are not a simple impulse or ramp. Thus, during one heartbeat, there was a wide variety in the signal, with rapid increases and decreases, as well as different magnitudes observed in our patients. How this complex signal variety can influence EC remodeling and gene expression remains an open question, which needs to be addressed in future studies. We can classify signals recorded from the 5 patients into two groups: (1) patients 1 and 5 with two WSSTD peaks at the beginning and end of the cycle and relatively constant values in-between; and (2) patients 2, 3, 4 with many random peaks during the whole cycle. This observation raises several questions: Is there a difference between these two groups regarding their influence on EC remodeling? Could one of these two groups reflect patients who develop atherosclerosis without any classical risk factors, such as smoking, fat diet or hypertension?

An important question relates to which values can be set as trigger or threshold values to influence EC functions? In a review [8], three conditions were proposed: (1) WSSTD = 0 Pa/s as steady condition; (2) WSSTD = 7.1 Pa/s as low condition; and (3) WSSTD = 29.3 Pa/s as high condition. When these conditions were applied to our data (Fig. 8), close to 90% of the WSSTD for patient 1 were between −7 and 7 Pa/s, 41.9% for patient 2 and about 60% for the other patients. Furthermore, almost 10% of WSSTD were in the range of high values for patient 2; this was about two times less than for the other patients. However, that high condition i.e. WSSTD about 29.3 Pa/s appears in a fraction of time. It seems that they are placed at higher frequencies of the signal. This triggers another question: Is it enough to activate ECs function with high frequency and high amplitude stimuli? Feaver et al. [25], demonstrated how the frequency spectrum of the wall shear stress signal can regulate the inflammation including NF-kB activity. By modifying or removing particular frequency harmonics from a carotid wall shear stress signal, they analyzed in vitro the response to human endothelial cells. They found that the frequency spectrum, specifically the 0th and 1st harmonics, is a significant regulator of inflammation. However, is it valid to the whole mechanisms of shear stress mechanotransductions? In the future study, in addition to the mentioned signals stimuli, the spectrum analysis of the WSS and WSSTD signal to the ECs will be considered.

This short analysis shows that it is difficult to agree on a constant value and that the patterns of variability in different patients are considerable. Nevertheless our study in 5 patients provides a limited set of preliminary data to plan in vitro experiments and create a database of patient flow patterns.

The aim of this project was also to provide bioengineers and physicians with a tool that will be able to mimic blood flows and later automatically calculate desired flow properties, such as WSS and WSSTD.

These calculations of the real coronary wall shear stress signals are critical for future development. The challenge will now be to reproduce these signals perfectly in this in vitro system. Meeting this challenge (see above) will also allow two critical observations made in this paper to be exploited: patients can be divided into different groups with different behaviors, which are much more complex than described in previous publications. These observations in turn raise two critical questions: Which of these behaviors is important in triggering the EC pro-atherogenic genes? Do these different behaviors explain why many patients with no risk factors develop a clinical condition and some patients with risk factors do not?

## Conclusions

The presented post-processing method is compatible with any in vivo signals and demonstrated the heterogeneity among patients in the pulsatile pattern in coronary arteries. In addition, we provide a new test bench able to reproduce human coronary blood flow for in vitro experiments.

## Abbreviations

BMS: bare metal stent
CFD: computational fluid dynamics
CFR: coronary flow reserve
C_n_: complex Fourier coefficient
D: diameter (m)
DES: drug eluting stent
ECs: endothelial cells
FFR: Fractional Flow Reserve
Hct: hematocrit (%)
LAD: left anterior descending artery
LCX: left circumflex artery
PCI: percutaneous intervention
QCA: quantitative coronary angiography
WSSTD: wall shear stress time derivative (Pa/s)
WSS: wall shear stress (Pa)
C_n_: complex Fourier coefficient
D: diameter (m)
G_n_: pressure gradient amplitude (Pa/m)
J_0_, J_1_: Bessel function of order 0 and 1 (–)
L: length (m)
n: nth component of a Fourier decomposition
p: dimensionless coefficient
P: pressure (Pa)
Q: flow rate (m^3^/s)
R_1_,r: radius (m)
Re: Reynolds number (–)
t: time (s)
U_z_: velocity in the axis direction z (m/s)
Λ: dimensionless number
Ω: dimensionless Womersley’s number
Δ: period (s)
$$\dot{\gamma }$$: shear rate (s^−1^)
$\mu_{0}$: dynamic viscosity at zero shear rate (Pa s)
$\mu_{\infty }$: dynamic viscosity at infinite shear rate (Pa s)
λ: relaxation time parameter (s)
$$\zeta$$: dimensionless number
$\mu$: dynamic apparent viscosity (Pa s)
$\tau$: shear stress (Pa)
$$\omega$$: pulsation (rad/s)
ρ: density (kg/m^3^)
